# Utilization of bioimpedance spectroscopy in the prevention of chronic breast cancer-related lymphedema

**DOI:** 10.1007/s10549-017-4451-x

**Published:** 2017-08-22

**Authors:** David I. Kaufman, Chirag Shah, Frank A. Vicini, Marisa Rizzi

**Affiliations:** 1 Breast Care Specialists, Bethpage, NY USA; 2 Department of Radiation Oncology, Cleveland Clinic, Taussig Cancer Institute, Cleveland, OH USA; 3 Michigan Healthcare Professionals, 21st Century Oncology, Farmington Hills, MI USA

**Keywords:** Breast cancer, Lymphedema, Bioimpedance spectroscopy, Survivorship, Toxicity, L-Dex

## Abstract

**Background:**

This analysis was performed to assess the impact of early intervention following prospective surveillance using bioimpedance spectroscopy (BIS) to detect and manage breast cancer-related lymphedema (BCRL).

**Methods:**

From 8/2010 to 12/2016, 206 consecutive patients were evaluated with BIS. The protocol included pre-operative assessment with L-Dex as well as post-operative assessments at regular intervals. Patients with L-Dex scores >10 from baseline were considered to have subclinical BCRL and were treated with over-the-counter (OTC) compression sleeve for 4 weeks. High-risk patients were defined as undergoing axillary lymph node dissection (ALND), receiving regional nodal irradiation (RNI), or taxane chemotherapy. Chronic BCRL was defined as the need for complex decongestive physiotherapy (CDP).

**Results:**

Median follow-up was 25.9 months. Overall, 17% of patients had one high-risk feature, 8% two, and 7% had three. 9.8% of patients were diagnosed with subclinical BCRL with highest rates seen following ALND (23 vs. 7%, *p* = 0.01). Development of subclinical BCRL was associated with ALND and receipt of RNI. At last follow-up, no patients (0%) developed chronic, clinically detectable, BCRL. Subset analysis was performed of the 30 patients undergoing ALND. Median number of nodes removed was 18 and median number of positive nodes was 2. 77% received taxane chemotherapy, 62% axillary RT, and 48% had elevated BMI. Overall, 86% of patients had at least one additional high-risk feature, 70% at least two, and 23% had all three. Seven patients (23%) had abnormally elevated L-Dex scores at some point during follow-up. To date, none has required CDP.

**Conclusions:**

The results of this study support prospective surveillance utilizing BIS initiated pre-operatively with subsequent post-operative follow-up measurements for the detection of subclinical BCRL. Intervention triggered by subclinical BCRL detection with an elevated L-Dex score was associated with no cases progressing to chronic, clinically detectable BCRL even in very high-risk patients.

## Introduction

Breast cancer represents the most common non-cutaneous malignancy for women in the United States with over 250,000 new cases diagnosed each year. With improved outcomes, the number of breast cancer survivors is growing dramatically [1, 2]. As a result, a significant number of women are dealing with the potential acute, sub-acute, and chronic toxicities of treatment including breast cancer-related lymphedema (BCRL). BCRL can be a minor aggravation for some patients or a life-altering side effect of treatment causing significant impairment of quality of life in others [3]. BCRL is unique in that it has an acute/sub-acute reversible phase and a more chronic, irreversible stage as it progresses [4–6].

The incidence of BCRL is highly dependent on the treatment paradigm utilized to treat a patient’s stage of disease and can be significantly impacted by more aggressive locoregional therapy (mastectomy, axillary dissection, regional nodal irradiation) or certain systemic chemotherapy agents [4, 7–9]. Additionally, patient factors such as body mass index (BMI) may also impact its incidence [10]. Increasingly, data and guidelines support the use of prospective surveillance programs to detect BCRL in its subclinical (reversible) phase to allow for early conservative treatment in order to prevent progression to its chronic and costly, irreversible phase [11–14]. However, screening all patients for the development of BCRL has proven difficult secondary to logistical and cost-related issues. As a result, it is useful to identify which patients are at highest risk to develop BCRL so that they can be targeted and enrolled in prospective surveillance programs.

Ideally, screening programs should utilize tools that allow for the detection of subclinical BCRL, an entity that is difficult to identify with traditional techniques [4, 7]; Bioimpedance spectroscopy (BIS) represents a relatively newer technique with the ability to detect subclinical BCRL in the clinic with limited time, personnel and space requirements [15–18]. Although there are numerous studies supporting the value of prospective surveillance with BIS, additional data are useful to further validate and refine its use in BCRL screening programs.

The purpose of the current study was to report the results of a large, structured surveillance program for BCRL utilizing BIS in all patients, many of which were considered high-risk. The rates of both subclinical and chronic BCRL were analyzed (for comparison with rates reported in historical controls of similarly treated patients) as well as factors associated with their development.

## Materials and methods

Between August 6, 2010 and December 14, 2016, 206 patients were evaluated as part of a prospective BCRL surveillance program at a single institution. All patients underwent measurements using the L-Dex U400 Device (ImpediMed, Brisbane, Australia). Inclusion criteria included patients with breast cancer undergoing definitive breast cancer surgery (breast conservation or mastectomy) with no limitation on the axillary management technique. Exclusion criteria included implantable electronic devices (i.e., pacemakers), pregnancy, renal failure, and heart failure. Patient characteristics, tumor characteristics, and treatment factors (surgery, axillary technique, radiation therapy, endocrine therapy, and systemic therapy) were documented (see below). Institutional review board (IRB) approval was provided for this retrospective analysis (WIRB Exemption Determination under 45 CFR 46.101(b)(4)).

Patients were followed prospectively using a standardized procedure. In brief, patients underwent a pre-operative baseline L-Dex measurement, then post-op follow-up measurements at 6 weeks, then 3–6 month intervals. The L-Dex measurement technique was based on previously published protocols [18]. With respect to defining subclinical BCRL, an elevated L-Dex score >10 from baseline was used as a cut-off criterion. In patients meeting this threshold, treatment was triggered with the use of an over-the-counter (OTC) compression sleeve for 4 weeks. Following treatment, patients underwent repeated L-Dex measurement to assess response. Patients not having resolution (as assessed by L-Dex) were defined as chronic BCRL and sent for complex decongestive physiotherapy (CDP).

Data collected for this analysis included patient characteristics (age and BMI) as well treatment characteristics (surgery, axillary management, chemotherapy, radiation therapy, and regional nodal irradiation). L-Dex scores were recorded at each visit as was documentation of BCRL treatment. For the purpose of this analysis, high-risk was defined as patients undergoing axillary lymph node dissection (ALND), receiving taxane chemotherapy, or regional nodal irradiation (RNI). Descriptive statistics are reported as mean (SD), median and range. Differences between groups were tested using Wilcoxon rank sum tests for quantitative variables and Chi-squared tests for categorical variables. Additional analyses performed evaluated the cohort of patients (*n* = 30) undergoing ALND. Analyses were performed using R version 3.2 or higher. A p value less than 0.05 was considered statistically significant.

## Results

Patient and treatment characteristics are presented in Table 1. Overall, 206 patients were evaluated with a mean age of 61 years. With respect to risk factors for BCRL, 45% of patients underwent mastectomy, 15% ALND, 28% of patients received chemotherapy (23% taxanes), and 19% received RNI. Of these factors (ALND, taxane chemotherapy, RNI), 17% of patients had only one factor present, 8% had two factors, and 7% had all three factors. Additionally, 50% of the cohort had an elevated BMI. Patients undergoing ALND were more likely to undergo mastectomy (70 vs. 41%, *p* = 0.006), receive chemotherapy (neoadjuvant [23 vs. 2%] or adjuvant [57 vs. 18%]), *p* < 0.0001), and receive RNI (63 vs. 12%, *p* < 0.001).

**Table 1:** Patient characteristics

	ALND	SLND	Total	*p*
*N*	30	176	206	
Age at baseline				0.132
Mean (SD)	58 (13.3)	61.3 (12.6)	60.8 (12.7)	
Median	57.5	62	62	
Range	38, 38	24, 89	24, 89	
BMI high	14 (48%)	83 (50%)	97 (50%)	1.000
Final type of surgery M	21 (70%)	72 (41%)	93 (45%)	0.006
Adjuvant Y	17(57%)	32 (18%)	49 (23%)	<0.001
Neo adjuvant Y	7 (23%)	3 (2%)	10 (5%)	<0.001
Taxane Y	23 (77%)	27 (15%)	50 (23%)	<0.001
Targeted (herceptin or TKI) Y	2 (7%)	7 (4%)	9 (4%)	0.855
WB Y	21 (70%)	87 (49%)	108 (50%)	0.059
Regional nodal irradiation Y	19 (63%)	22 (12%)	41 (19%)	<0.001
APBI Y	1 (3%)	19 (11%)	20 (9%)	0.346

*ALND* axillary lymph node dissection, *SLNB* sentinel lymph node biopsy, *BMI* body mass index, *TKI* tyrosine kinase inhibitor, *WB* whole breast irradiation, *APBI* accelerated partial breast irradiation

Median follow-up was 25.9 months for the entire cohort (range 3.6–116.7 months) and 35.6 months for patients undergoing ALND (range 3.6–122.1 months) as compared to 24.5 months for those undergoing sentinel lymph node biopsy (SLNB) (range 3.6–122.1 months) (*p* = 0.12). The median number of follow-up measurements was 7 for the entire cohort and 8 (5/7) for ALND/SLNB patients, respectively.

Overall, 9.8% (*n* = 21, 95% CI 6.2–14.5%) of patients were diagnosed with subclinical BCRL (increase in L-Dex >10) with 23% (*n* = 7) of ALND patients diagnosed as compared to 7% (*n* = 12) of SLNB patients (*p* = 0.01). Median time to diagnosis was 8.7 months and was 7/10.3 months for ALND/SLNB patients, respectively. Patient and treatment characteristics by status of BCRL development are presented in Table 2. Patients who developed subclinical BCRL were less likely to undergo SLNB (63 vs. 88%, *p* = 0.01) and more likely to have received RNI (38 vs. 17%, *p* = 0.04). Median follow-up for patients developing subclinical BCRL was 24.2 months with an increased median number of follow-up measurements (9 vs. 6, *p* = 0.09). No patients developed chronic, clinically detectable BCRL requiring CDP and had a median time to resolution of an elevated L-Dex score overall of 1.9 months and 4.3/1.4 months for ALND/SLNB patients, respectively, (No patient diagnosed with an elevated L-Dex score >10 (subclinical BCRL) and treated with an OTC sleeve for 4 weeks required CDP).

**Table 2:** Characteristics by development of BCRL

	*N*	Y	Total	*p*
*N*	194	21	215	
Age at baseline				0.297
Mean (SD)	60.5 (12.8)	63.6 (12)	60.8 (12.7)	
Median	62	65	62	
Range	24, 89	41, 88	24, 89	
BMI high	86 (49%)	11 (58%)	97 (50%)	0.613
Final type of surgery M	83 (45%)	10 (53%)	93 (45%)	0.670
Final type of axillary surgery SLNB	164 (88%)	12 (63%)	176 (85%)	0.011
Adjuvant Y	42 (22%)	7 (33%)	49 (23%)	0.348
Neo adjuvant Y	8 (4%)	2 (10%)	10 (5%)	0.568
Taxane Y	43 (22%)	7 (33%)	50 (23%)	0.379
Targeted (herceptin or TKI) Y	8 (4%)	1 (5%)	9 (4%)	1.000
WB Y	94 (48%)	14 (67%)	108 (50%)	0.175
Regional nodal irradiation Y	33 (17%)	8 (38%)	41 (19%)	0.041
APBI Y	20 (10%)	0 (%)	20 (9%)	0.250

*ALND* axillary lymph node dissection, *SLNB* sentinel lymph node biopsy, *BMI* body mass index, *TKI* tyrosine kinase inhibitor, *WB* whole breast irradiation, *APBI* accelerated partial breast irradiation

Among those patients undergoing ALND, the median follow-up was 36 months (range 4.8–122.1 months). The median age of the cohort was 57.5 years with 70% of patients undergoing mastectomy and the remainder breast conserving therapy. The median number of nodes removed was 18 (range 5–32), and the median number of positive nodes was 2. With respect to additional high-risk features, 77% received taxane-based chemotherapy, 62% regional nodal irradiation, and 48% had an elevated BMI. Overall, 86% of these patients had at least one additional high-risk feature, 70% at least two, and 23% had all three additional high-risk features. A total of 7 patients (23% of ALND patients) had an elevated L-Dex score and underwent intervention with an OTC sleeve. No patients required CDP.

## Discussion

The results of the current study demonstrate the importance of structured surveillance in reducing the impact of BCRL on women treated for breast cancer. Using serial BIS measurements, subclinical, reversible BCRL was detected in 9.8% of patients with no patients progressing to require CDP for chronic, clinically detectable BCRL. Although limited by short follow-up and small patient numbers, these results compare favorably to studies reporting BCRL rates in the range of 10–50% without the use of structured, prospective surveillance [19–24]. Even when compared to results observed in a contemporary, low-risk cohort of patients (as seen in the recent ACOSOG Z0010 trial where a 7% rate of clinical BCRL was noted at 6 months), results in the present study (composed of a higher risk cohort) compare quite favorably [25]. Combined with recently published prospective data (see Table 3) documenting the impact of early detection and treatment of subclinical BCRL in reducing the rate of chronic BCRL, these findings support the importance of structured surveillance programs in reducing the morbidity of BCRL.

**Table 3:** Prospective studies evaluating early detection and treatment to reduce rates of chronic BCRL

Institution	Year published	Number of patients	Measurement technique	Treatment	Results
University of Queensland	2002	65	Circumference + BIS	Physiotherapy vs. standard follow-up	Physiotherapy reduced BCRL at two years (11 vs. 30%)
National Naval Medical Center	2008	196	Perometry	Compression garment with detection	Only 6% of patients developed chronic BCRL
Alcala de Henares University	2010	120	Circumference	Physiotherapy vs. education	Physiotherapy reduced BCRL at one year (7 vs. 25%)
University of Pittsburgh Medical Center	2014	186	BIS	Compression garment with detection	Clinical BCRL 4.4% with BIS vs. 36.4% with circumferential measurement follow-up

### Defining a high-risk population

Despite the recommendations of the recent NCCN guidelines on Surveillance/Follow-Up after breast cancer treatment to educate and monitor patients for BCRL, defining a high-risk population of patients that can be more easily targeted seems justified. The data from the current study identified ALND and RNI as two factors associated with the development of subclinical BCRL. These observations are consistent with previous studies that also found these factors (along with taxane chemotherapy and an elevated BMI) to be associated with the development of BCRL [4, 7–9, 19–22, 25, 26]. Additionally, a recent study from Turkey which utilized BIS and circumference arm measurements found BMI, number of nodes involved, and capsular invasion to be associated with BCRL, with the number of positive nodes also correlating with the L-Dex score [27]. Many of these factors are treatment related and based on disease stage and as such cannot be omitted in the management of patients. Therefore, with the increasing use of multi-modality therapy in conjunction with improved clinical outcomes, targeted prospective surveillance represents an important strategy to improve quality of life and survivorship for high-risk patients. Additionally, by identifying high-risk cohorts, it avoids the need to screen all patients and therefore, is a more cost and resource effective option.

### Impact of early detection

One of the driving factors for the increased use of prospective BCRL surveillance programs is the growing body of data and recent guidelines supporting the concept that early detection and intervention not only allows for subclinical BCRL detection but also a reduction in the rates of chronic BCRL (Table 3). For example, Soran et al. prospectively evaluated 186 patients with BIS who had undergone ALND. Patients diagnosed with subclinical BCRL (increase of greater than 10 units from baseline or L-Dex scores outside of −10 or +10) were treated with a short-term compression garment, education, and physical therapy, while a control group had a single L-Dex measurement and were subsequently followed with only circumferential measurements. Within the BIS cohort, 33% of patients were diagnosed with subclinical BCRL with only 4.4% progression to chronic BCRL as compared to 36.4% in the control group [12]. This is consistent with data from Turkey (Iyigun et al.), which found that in 21% of cases, BCRL was detected by BIS and not circumference arm measurements [27]. While previous diagnostic techniques were limited in their sensitivity and ability to detect subclinical disease, recent advances have led to diagnostic techniques including BIS with increased sensitivity [15, 16]. Additionally, as compared to other modern diagnostic modalities (e.g., perometry), BIS has a small physical footprint, is portable, can be performed with minimal cost and time and has been found to result in equal outcomes [18]. While one of the initial prospective studies evaluating early detection and treatment used perometry, with updated data, it is clear that BIS may be an appropriate and equally effective alternative [14]. Additionally, growing data support changing the traditional three standard deviation BIS cut-off (L-Dex increase >10 from baseline) to two standard deviations, and thus allowing for further increase in the sensitivity to detect subclinical BCRL and a potentially greater reduction in the rate of chronic BCRL [28, 29].

### High-risk patients

As mentioned previously, although prospective surveillance for BCRL has been recommended by the NCCN as part of the routine follow-up for all breast cancer patients, it would seem logical to focus greater attention to the highest risk patients [30]. One of the most significant risk factors for the development of BCRL has been the use of ALND. Even in modern studies using ALND, the risk of BCRL ranges from 20 to 53%—and the risk increases with the number of nodes removed (see Table 4) [23, 31–35]. The addition of RNI RT can substantially increase this risk as can the use of taxanes and having a patient with an elevated BMI. In the subset of patients undergoing ALND in the current study (as discussed previously), the median number of nodes removed was 18, 77% of cases received taxane-based chemotherapy, 62% axillary irradiation, and 48% had an elevated BMI. Overall, 86% of ALND patients had at least one additional high-risk feature, 70% at least 2, and 23% had all three. With a median follow-up of 3 years, seven of these patients (23%) had an elevated L-Dex score at some point during follow-up and underwent intervention with an OTC sleeve for 4 weeks. Despite all these high-risk features, no patient has required CDP at any time. Although patients remain at risk for the development of BCRL many years after treatment, these results compare quite favorably to similarly treated patients reported in the modern literature.

**Table 4:** Rates of lymphedema in patients undergoing axillary lymph node dissection

	Lymphedema incidence	Follow-up	Diagnostic technique
NSAPB B32 [23]	14%	36 months	Water displacement
Denmark [31]	16–18%	39–51 months	Circumference measurement
University of Sydney [32]	18.2%*	18 months	Bioimpedance spectroscopy
University of Pittsburgh [33]	12.2%	21 months	Tape measure
Italy [34]	27%	50 months	Circumference measurements
AMAROS [35]	13/23%	60 months	Arm circumference/clinical signs
Present Study	0%	36 months	Bioimpedance spectroscopy

* >5 nodes removed

### Study limitations

There are limitations to the present analysis. While data were collected prospectively as part of a surveillance program, this was a retrospective review and, as such is associated with the limitations (potential biases) of such an analysis. Additionally, follow up was relatively short (25 months mean), limiting analyses of long-term outcomes. Also, the total number of events was small, limiting the ability to compare factors associated with subclinical BCRL diagnosis. However, these data add to the growing body of literature supporting prospective BCRL surveillance in high-risk patients to allow for early detection and treatment.

Future studies are expected to provide long-term follow-up outcomes with such an approach. In the interim, this model should be considered as an option as part of breast cancer survivorship programs. Fortunately, a prospective randomized trial is underway comparing BIS with circumference tape measurements to determine the magnitude of the reduction in the rate of chronic BCRL with structured surveillance and early intervention [36]. Breast cancer patients (*n* = 1100) with at least one risk factor for the development of BCRL are enrolled and then monitored with either BIS or circumference measurements pre-operatively and at 3,6,12,18,24, and 36 months. Patients with an L-Dex increase of ≥5.5 undergo circumference arm measurements and then treatment with an OTC compression sleeve for 4 weeks, while those with a volume increase of 5–10 cc will undergo L-Dex testing and compression sleeve for 4 weeks. The primary endpoint of the study is the rate of progression of BCRL (requirement for CDP) with the hypothesis being that prospective surveillance with BIS allows for early intervention, reducing the rate of chronic BCRL. Results are expected in the next few years.

In summary, BIS represents a valuable and practical tool for the early detection of subclinical BCRL in patients undergoing prospective monitoring. In this prospective surveillance study, use of BIS allowed for early intervention and a reduction in the predicted rate of chronic BCRL compared to historical controls (no cases of persistent, chronic BCRL were observed after early intervention even in the highest risk patients). Such an approach represents not only a valuable strategy to address the recent NCCN guidelines on survivorship for monitoring for BCRL but also a cost-effective strategy to prevent and manage the potentially devastating effects of chronic BCRL.
